# Supplementary material for: The interactome of intact mitochondria by cross-linking mass spectrometry provides evidence for coexisting respiratory supercomplexes
Source: Mol Cell Proteomics. 2017 Dec 8;17(2):216–32. doi: 10.1074/mcp.RA117.000470 (PMC5795388; doi:10.1074/mcp.RA117.000470)
Supplement: Supplemental Data [file supp_RA117.000470_133922_0_supp_23978_fzffwf.zip › spectra_annotation/mito_spectra_annotation/356-2-12-1-2-1.pdf]

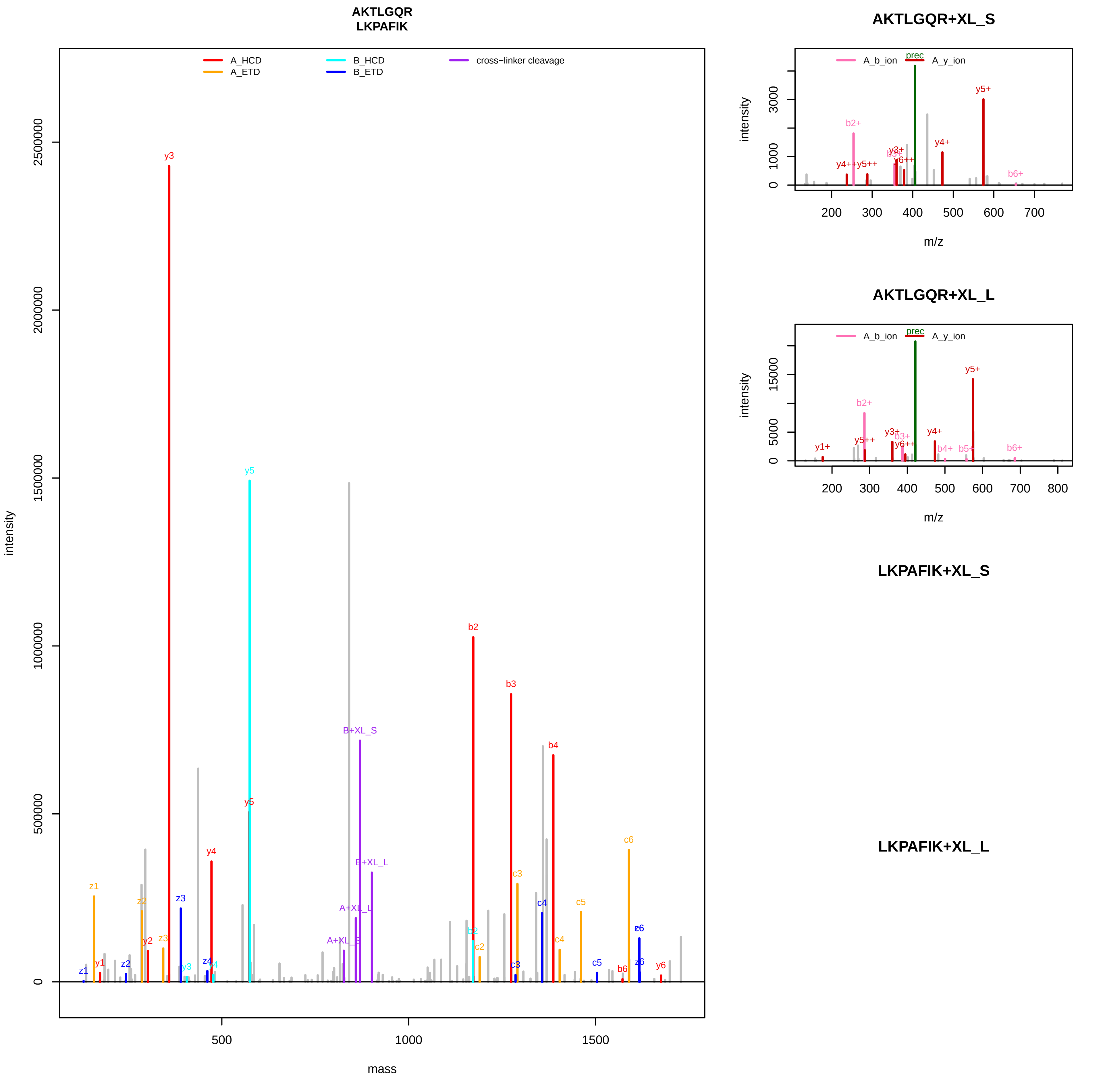
AKTLGQR
LKPAFIK
A_HCD
A_ETD
B_HCD
B_ETD
cross−linker cleavage
intensity
mass
0
500000
1000000
1500000
2000000
2500000
500
1000
1500
y3
y5
b2
b3
b4
B+XL_S
B+XL_L
A+XL_L
A+XL_S
y4
y5
z1
y1
z2
y2
z3
y3
z4
y4
c2
c3
c4
c5
c6
z6
c6
z6
b6
y6
AKTLGQR+XL_S
A_b_ion
A_y_ion
prec
y5+
b2+
y4+
y4++
y5++
y3+
b3+
y6++
b6+
intensity
m/z
AKTLGQR+XL_L
A_b_ion
A_y_ion
prec
y5+
b2+
y4+
y3+
y5++
b3+
y6++
y1+
b4+
b5+
b6+
intensity
m/z
LKPAFIK+XL_S
LKPAFIK+XL_L
